# Design and implementation of a health messaging protocol employed for use within a COVID-19 health dissemination platform

**DOI:** 10.3389/fpubh.2022.942795

**Published:** 2022-11-24

**Authors:** Paulina M. Colombo, Sarah Freylersythe, Mary Margaret Sprinkle, Kacey C. Ernst, Marcela Yubeta, Juliana L. Barbati, Nirav Merchant, Sriram Iyengar, Tracy E. Crane, Maliaca Oxnam, Stephen A. Rains

**Affiliations:** ^1^Department of Epidemiology and Biostatistics, College of Public Health, University of Arizona, Tucson, AZ, United States; ^2^The University of Arizona Cancer Center, Tucson, AZ, United States; ^3^Department of Internal Medicine, University of Arizona College of Medicine, Phoenix, AZ, United States; ^4^BIO5 Institute, The University of Arizona, Tucson, AZ, United States; ^5^Department of Communication, The University of Arizona, Tucson, AZ, United States; ^6^Data Science Institute, The University of Arizona, Tucson, AZ, United States; ^7^Sylvester Comprehensive Cancer Center, Miller School of Medicine, University of Miami, Miami, FL, United States

**Keywords:** health messaging, health education (MeSH), short message services (SMS), COVID-19, SARS-CoV-2, pandemic response

## Abstract

**Introduction:**

AZCOVIDTXT, a bilingual, two-way information sharing platform was created in April of 2020 in response to rising COVID-19 cases in Arizona. The aim of this paper is to delineate the protocol and processes used to develop and disseminate health messaging to serve as guidance for other groups, universities, or public health programs in the implementation or enhancement of health communication services.

**Methods:**

Health messaging formats included website articles, published on the system's website (azcovidtxt.org), infographics posted on social media, and SMS. Social media and SMS infographics were intended to highlight and augment the topics covered in the weekly website articles, to create a seamless multimodal source of reliable COVID-19 information for AZCOVIDTXT enrollees and the broader public. All health messaging information, text message and social media content was planned and reviewed collaboratively by the AZCOVIDTXT team topic experts for accuracy, efficacy, and content consistency.

**Results:**

As of July 2021, AZCOVIDTXT provided weekly COVID-19-related health communication to 3,747 participating households located across 225 Arizona zip codes. AZCOVIDTXT has developed and sent 446 unique, bilingual SMS for a total of 271,977 contact points. The team has produced and published 179 website articles, which averaged a combined 7,000-page views per month, and 173 social media posts were made available to 268 followers across three platforms.

**Discussion:**

Several programmatic aspects were deemed essential to the success of AZCOVIDTXT. These included (1) addressing community specific needs, (2) creating timely and relevant content, (3) developing an adaptable system, and (4) prioritizing system automation where possible, (5) having an interdisciplinary team approach to identifying and crafting key messages.

## Introduction

Since the start of the COVID-19 pandemic, an abundance of misinformation (1–3), rapidly changing science, and a lack of access to reliable COVID-19 information has hindered United States mitigation efforts (4). Ambiguous and contradictory health recommendations also lead to significant losses of the public's trust in governmental organizations and science (5). Researchers at the University of Arizona identified a critical need to create a mutually beneficial platform to (1) provide local Arizona residents' with access to critical, continually evolving COVID-19 information and (2) assist scientists in collecting COVID-19 data. A multidisciplinary group of data science, public health, behavioral science, health education, and communication experts developed a service to accomplish these goals.

AZCOVIDTXT, a bilingual, two-way information sharing platform was created in April of 2020 in response to rising COVID-19 cases in Arizona, coupled with the spread of inaccurate information about the virus, which remain ongoing challenges today. The overarching goals for AZCOVIDTXT were outlined in a previously published article (6). We aim to delineate the protocol and processes used to develop and disseminate health messaging for AZCOVIDTXT as guidance for other groups, universities, or public health programs in the implementation or enhancement of health communication services. Our objective of rapidly deploying AZCOVIDTXT to meet the immediate and evolving needs created by the pandemic led us to not prioritize theory testing or formal evaluation protocols as might be the case in a traditional intervention. As such, we believe that our project offers a novel case study that can aid in preparing for and responding to unanticipated future health crises. This paper provides lessons learned from the methods used to implement and operate AZCOVIDTXT that drew upon the expertise of interdisciplinary team members. From April 2020 to July 2021, the AZCOVIDTXT team sent over 271,977 text messages, disseminated 179 articles, and created 173 social media posts.

## Materials and methods

### AZCOVIDTXT system overview

AZCOVIDTXT is a free service that Arizonans could voluntarily join at any time by sending a text message (SMS) to enroll. The system consists of two components which work in tandem to assist public health and Arizona residents in better understanding COVID-19. A surveillance component collects initial household demographic information, using the Research Electronic Data Capture (REDCap), from subscribers along with a brief, weekly survey where they can disclose household health, vaccination status, and challenges related to financial, social, or other issues encountered in the past week (7, 8). These surveys enable the second system component, a health messaging program, to be tailored to specific groups of respondents, such as those who report a sick household member. Subscribers can opt in to receive additional health communications in the initial demographic survey. This messaging component of the system provides regular, credible, Arizona-specific pandemic resources and information through weekly SMS, social media posts, and website articles in both English and Spanish. Multiple channels (e.g., website forum, email communications, SMS responses to outgoing messages, and a periodic community survey) for user feedback were developed to inform content creation and prioritize community interests. AZCOVIDTXT remains ongoing, but this article focuses on processes utilized from its establishment to July 2021. We next detail our process of content development and data management.

### Health messaging overview

Health messaging for AZCOVIDTXT was designed to meet the evolving information needs of Arizonans since the beginning of the COVID-19 pandemic. Health messaging modalities included SMS, website articles, published on the system's website (azcovidtxt.org), and infographics posted on social media. Research has demonstrated an increased effectiveness of health communication campaigns and improved adoption of health-related information when multiple media approaches are utilized (9). The unique affordances of each modality were leveraged to deliver complementary information about COVID-19 to a wide audience.

### Text messages (SMS)

Three to five SMS per week were determined to be ideal to avoid undue burden on enrollees (10). One weekly SMS, sent to all users, served a reminder to complete the brief weekly survey. This SMS contained a specific-participant link that was associated with the individual user's prior longitudinal data. Additional informative SMS were released with the goal of disseminating educational material to users. To receive this message type, subscribers opted-in based on their preferences during the AZCOVIDTXT enrollment process. Content for informational SMS was intended to mirror and augment website article material, and address challenges and topics of particular interest to Arizonans. One informative SMS sent to all subscribers was intended to spark interest and encourage subscribers to visit the weekly updated website articles. Messages that directed subscribers to view the website articles included a URL as part of the character count limit. A second informative SMS sent to all subscribers provided an opportunity to send more general information, such as current events and changes in official guidance, local Arizona supportive services, and social support reminders. All SMS were translated into Spanish by staff at the University of Arizona Cancer Center's Behavioral Measurements and Shared Resource (BMISR) and were released to subscribers who indicated a Spanish preference for receiving their informative SMS.

Data provided by the surveillance component of AZCOVIDTXT were reviewed to allow responsive messaging back to the users. This design was in accordance with research demonstrating higher efficacy for text messaging-based health promotion interventions when applying personalized messaging (11). Thus, these general SMS were often tailored, especially during the early months of the pandemic, to address the challenges that enrollees were reporting on their weekly surveillance surveys (e.g., accessing cleaning and hygiene supplies, medical and mental health care, and basic human services such as housing, food, and water). A third informative SMS was sent only to a specific group of subscribers: those who reported that someone in their household that week was sick. This group of messages included information specifically tailored to minimizing spread within households, how and when to isolate and quarantine, and when and how to seek urgent care.

AZCOVIDTXT offers real-time response. In the event of a critical or emergent issue, additional, one-time SMS could be sent. Examples of critical or emergent issues of public health and safety, included wildfire evacuations during lockdown, executive orders such as the Stay-at-Home order, and to address widespread and life-threatening misinformation. Urgent messages were only sent on six instances during 2020 (Table 1).

**Table 1:** Urgent SMS sent.

**English SMS sent**	**Date**
Drinking or injecting disinfectants will make you sick and can kill you. Use only as directed. Contact Poison Control immediately if needed: 800-222-1222	4/24/20 11:00 AM
From AZCOVIDTXT: The AZ Governor has extended the stay at home order for COVID-19 until May 15 in Arizona	4/29/20 4:30 PM
If you are evacuating, be sure to take masks and disinfecting supplies with you. Masks can reduce smoke particulate exposure AND risk of transmitting COVID-19	6/11/20 4:00 PM
Tucson Area Alert: Imminent fire danger in the Catalina Foothills area. Sign up for alerts from Pima County here: https://emergencyalerts.pima.gov/	6/11/20 4:00 PM
Maricopa County Alert: Imminent fire danger in the Tonto Nat'l Forest area. Stay up to date with state fire agencies: http://fb.com/bushfireinfo	6/19/20 2:00 PM
If you are evacuating, be sure to take masks and disinfecting supplies with you. Masks can reduce smoke particulate exposure AND risk of transmitting COVID-19	6/19/20 2:00 PM

SMS were sent *via* the AZCOVIDTXT communications platform: a Django-powered application developed by programmers and data scientists at the University of Arizona's Data Science Institute. The AZCOVIDTXT platform interfaced with Twilio, a cloud-based communication service, in order to send and receive SMS with subscribers (12). The AZCOVIDTXT communications platform enabled advanced scheduling of SMS for a specific date and time and to unique subscriber groups, such as Spanish SMS sent to Spanish-speaking enrollees. Further filtering was possible (e.g., by zip code or specific flags such as subscribers who reported a sick household member on a weekly survey), allowing for messages to be tailored as needed, (e.g., sending messages regarding wildfire evacuations to only the zip codes affected). The AZCOVIDTXT SMS communication platform also logged and tracked all incoming and outgoing SMS for reporting purposes. SMS released through this platform had a 160-character limit.

### Website articles

An average of three articles regarding pressing COVID-19 related information were developed and published on the system's website each week. The articles were complementary to the service's other communication features and served to provide comprehensive COVID-19 health information. The intent was to clarify current science and policies and provide tips and information for resilience during the pandemic. Specific topics included in the articles discussed an array of pandemic-related information including evolving COVID-19 science, state-issued executive orders, available Arizona resources, and interviews with field experts (e.g., university researchers, healthcare professionals, etc.). Articles typically ranged between 300 and 600 words and were written in a manner that could be easily interpreted by the lay public.

Decisions about the content included in each article were informed through multiple channels including (1) surveillance data input from users, (2) community feedback forms administered periodically, (3) feedback forum, email, and incoming SMS responses, and (4) as guided by AZCOVIDTXT team leads who had extensive backgrounds in health communication, public health, nursing, and health education prioritizing and ranking important emerging topics. The articles were mainly researched and written by AZCOVIDTXT staff who have expertise in public health and health education. Periodically, external health professionals and experts were consulted for additional content matter. After the material was drafted, any health-related content was carefully vetted by public health and nursing professionals as well as a health communication expert who refined language and formatting to eliminate any messaging inefficiencies. The content was then translated into Spanish, again by BMISR staff.

Individual articles were organized and titled using specific taglines (e.g., All Things Vaccines, Testing Tips, etc.) into 15 macro-categories. Overarching topic groups allowed readers to quickly identify the general content of an article prior to reading. These 15 article categories were collapsed into six dropdown menu items, which were made available in the resources tab on the main page of the website.

The AZCOVIDTXT website was a supplemental feature to the regular text messages received by subscribers of the platform. In addition to housing the weekly articles, the website provided general information about the program and team, and contained an automated, interactive map and corresponding table that displayed de-identified AZCOVIDTXT household subscriber data at the county and zip code level (e.g., confirmed case counts (gathered from Arizona Department of Health Services), the number of households reporting to AZCOVIDTXT and, of these, how many reported experiencing food scarcity). COVID-19 content could be easily located under the resources tab. Updated articles and social media material were condensed into 6 topic categories: High-Risk Groups (e.g. Kids, Essential Workers, Vulnerable Populations); COVID-19 Science; Prevention and Planning; State-Wide Mandates; Mental Health; and Vaccine Information. Additional relevant resources including volunteer and further research opportunities are also found under the website's resource tab.

### Social media infographics

To support and more broadly disseminate reliable COVID-19 information, infographics were developed weekly and posted on three major social media platforms: Twitter, Instagram, and Facebook. Information from the weekly website articles were adapted into an infographic with a focus to share the core ideas in a visual format. These visualizations were used as a tool to help process and retain complex or emerging COVID-19 updates quickly and clearly. Infographic messaging was intended to be brief, easy to read and understand, and eye catching. As of July 2021, AZCOVIDTXT had a combined follower count of over 350 across all three social media platforms.

Each week, ~2–3 infographics were developed. A brief description of the infographic was included as text with the post, and a wide variety of COVID-19, University of Arizona, and accredited public health entities (e.g. CDC, WHO, etc.) were tagged. Most infographics were a single picture, optimized for both desktop and mobile viewing. On Instagram, additional infographic sequences were developed to take advantage of the swipe-through carousel feature. All infographics were branded with the AZCOVIDTXT logo. Infographics were made available in English and Spanish.

The infographics were designed using Canva Pro, a web-based graphic design tool, and presented to the leadership team for review. After feedback was incorporated and all revisions made, the infographics were posted to all three social media platforms using the web-based platform SalesForce.

As a method of further outreach to the community, images were made available in a downloadable format for public access and so they could be repurposed for use by other organizations. This was beneficial for less-resourced, external groups who did not have the bandwidth or staffing to develop Arizona-centric graphics on their own. The team reached out to a range of community groups to promote these graphics as free and trustworthy resources including medical facilities, school districts, fitness centers, and restaurants.

### Content development and data management

**Figure 1:**
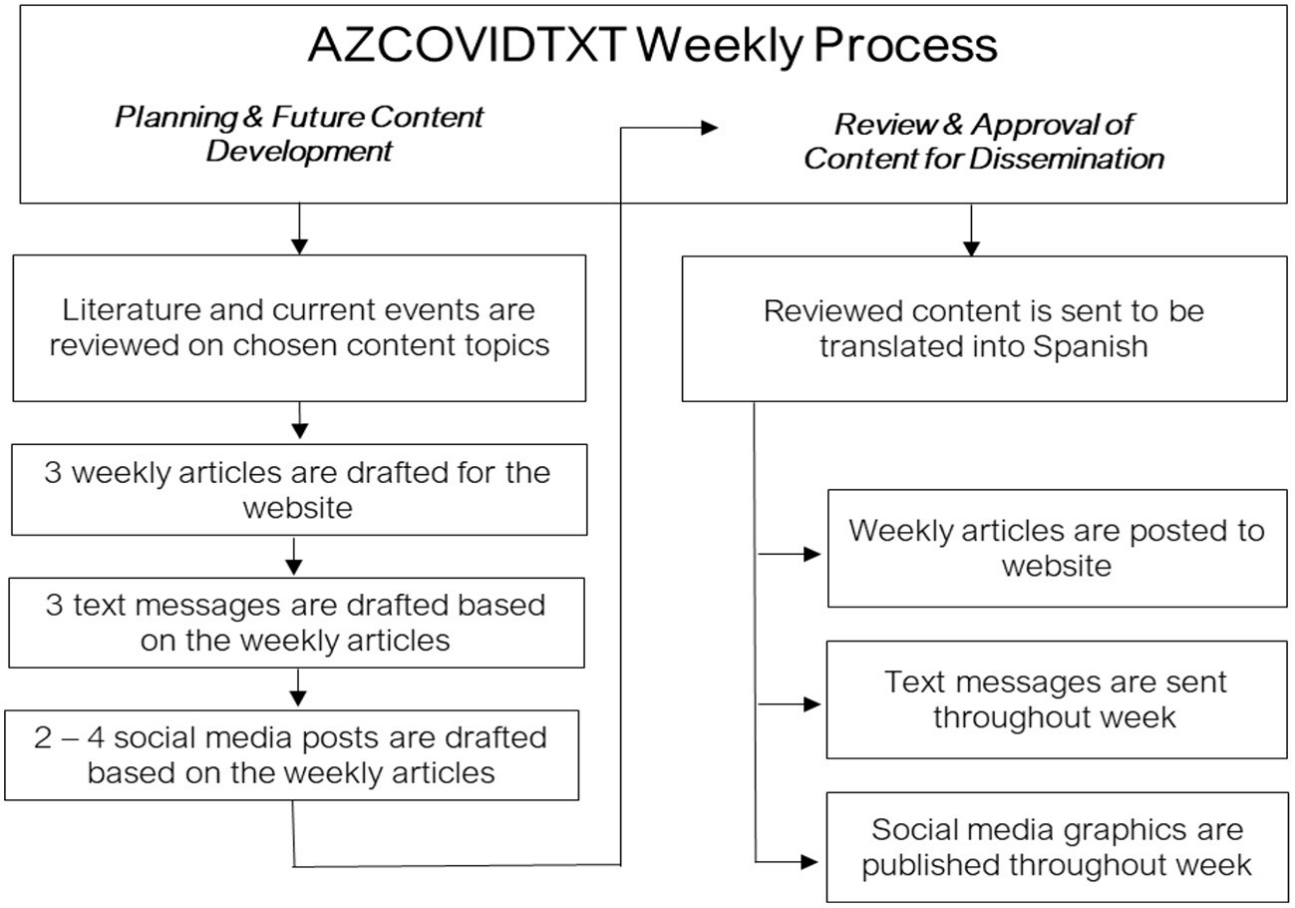
Content development and dissemination process outline.

The AZCOVIDTXT team met weekly in order to review and approve the following week's content (i.e., website articles, text messages, and social media infographics) and plan for future content. Developed content was reviewed by the team, revisions were made, and then the content was translated to Spanish. After identifying topics for the next week, the topics were researched, and the next round of content was drafted. Figure 1 delineates the content development and dissemination process.

### Community feedback evaluation

Providing tailored, Arizona-centric COVID-19 information was a fundamental goal of AZCOVIDTXT. Thus, acquiring community feedback was essential to incorporating and addressing the COVID-19-related concerns of local, participating Arizona residents. One optional program evaluation, provided in both Spanish and English, was administered in September 2020 and remained open to collect responses for 1 month. We chose to solicit responses approximately 5 months after beginning in April because we had accumulated a relatively sizeable number of subscribers and recognized that there would likely be a continued need for our service as the pandemic evolved. The survey was designed to evaluate our performance through anonymously collecting input regarding user preferences for content as well as to provide an opportunity for subscribers to voluntarily disclose confusion or worry about particular COVID-19 topics. Multiple question formats including multi-select and write-in options to allow for comprehensive feedback. A brief report containing both qualitative and quantitative survey data was then made available to subscribers through the platform's website. The results have also been regularly used to inform decisions regarding AZCOVIDTXT content output in the form of text messages, social media posts, and weekly articles posted on the platform's website.

## Results

In this section, we report on the user data that our service collected from April 2020 until July 2021. We also provide findings from the community feedback we conducted in September 2020.

### Subscriber base

Subscriber demographics are provided in Table 2. In brief, as of July 2021, AZCOVIDTXT provided weekly COVID-19-related health communication to 3,747 participating households located across 225 Arizona zip codes. Designated individuals who reported household health information were predominantly female (73.4%), and white (87.1%). 15.3% of household respondents identified as either Hispanic or Latinx. The average age of household respondents was 51.3 years (range = 15–99 years old, standard deviation = 15.9). The majority of enrollees opted in to receive the informational text messages (*n* = 1,940, 51.8%).

**Table 2:** Demographic characteristics of AZCOVIDTXT users (April 2020–July 2021), *n* = 3,337.

	**n**	**%**
**Gender**		
Male	864	25.9
Female	2,443	73.4
Non-binary	10	0.3
Other	2	0.1
**Age**		
15–29	385	11.6
30–44	766	23.0
45–59	962	28.9
60–74	1,040	31.2
75–99	176	5.3
**Are you Hispanic or Latino/a?**		
No	2,769	84.7
Yes	501	15.3
**Race**		
American Indian or Alaska Native	49	1.5
Asian	94	2.9
Black or African American	41	1.3
Native Hawaiian or Other Pacific Islander	11	0.3
White	2,801	87.1
More than one race	116	3.6

Column headers may not sum to table total due to missing data.

### Content and engagement

**Figure 2:**
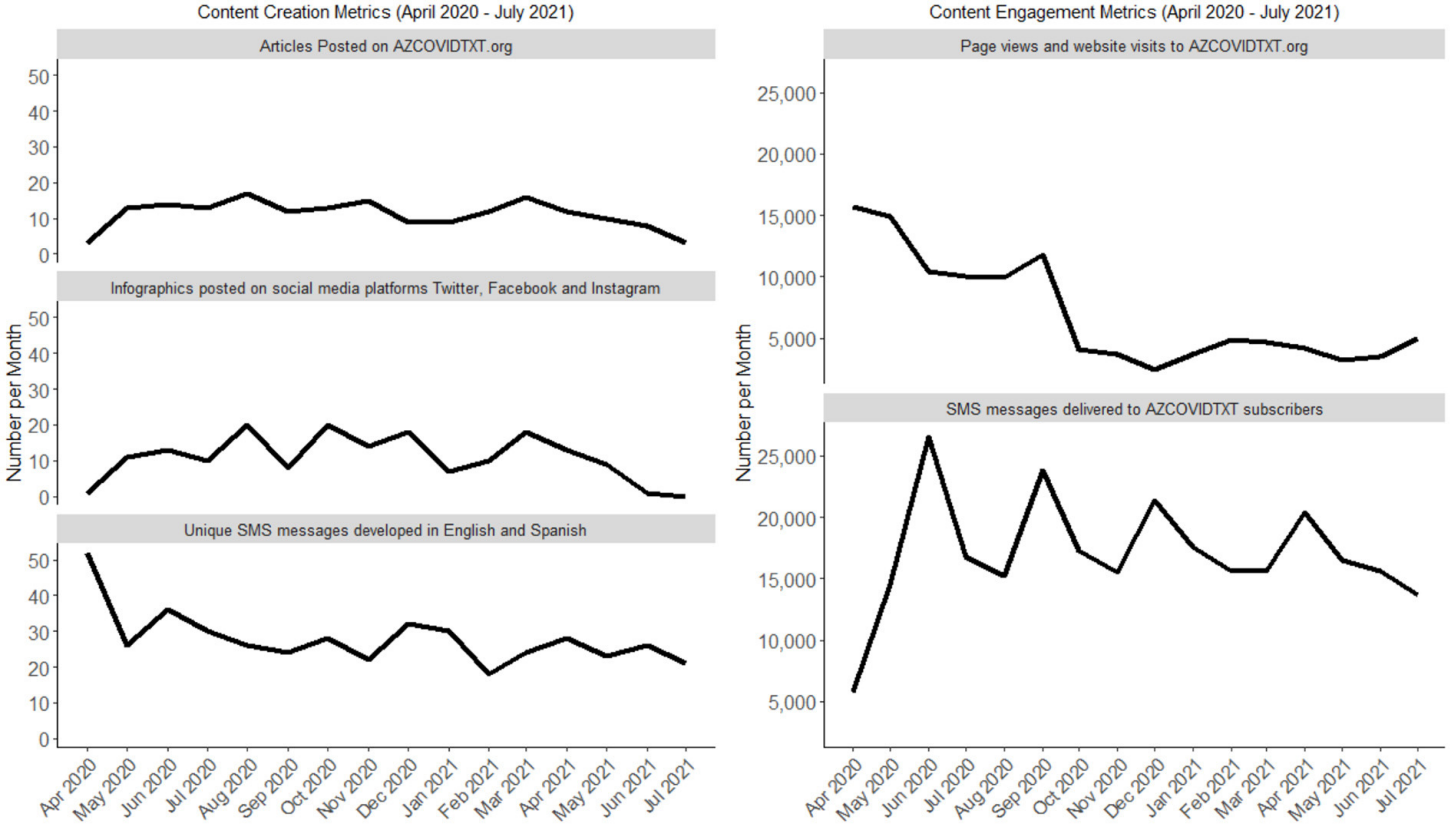
Content creation and engagement metrics.

AZCOVIDTXT has developed 446 unique SMS in two languages, English and Spanish, and sent a total of 271,977 SMS to subscribers. The team has produced and published 179 website articles in both English and Spanish. From April 2020 to July 2021, the weekly website articles averaged 7021 views per month (SD: 4410.5; 95% CI: 4670.7, 9371.1), and the AZCOVIDTXT website received 112,334-page views as a whole. Additionally, 173 social media posts were developed and made available to 268 combined followers across three platforms. Figure 2 provides content creation and engagement metrics of the platform's different modes of communication. There was a gradual decrease in website page views as the pandemic persisted, which may be due to pandemic fatigue.

### Community feedback

The community feedback evaluation yielded responses from over 450 subscribers. To inform content creation, users were asked to provide COVID-19 topic areas that were of most interest to them. The most common responses focused on the long-term health impacts of COVID-19 and vaccine safety and effectiveness. Respondents also overwhelmingly noted concern regarding an overall lack of precautionary measures taken in their communities and a mistrust of politicized COVID-19 information.

## Discussion

### Successes

The AZCOVIDTXT team successfully developed and implemented a protocol to disseminate COVID-19 content to more than 3,700 households located across 225 zip codes over the first 16 months of the pandemic. AZCOVIDTXT's large subscriber base emphasized the need for an information platform to provide timely and credible information about COVID-19 specific to the citizens of our state. Moreover, the sustained enrollment in the SMS system component suggested subscriber satisfaction with the program.

The multidisciplinary team provided access to expert reviewers across many fields of study. Further, the large majority of funding for this work supported personnel time for website and texting service implementation, content development, and review by public health experts. Thus, the project provided an opportunity to involve students from journalism, public health, and public relations to assist with operational areas of the project.

### Challenges and limitations

As AZCOVIDTXT developed highly dynamic and responsive material, the development process required weekly planning meetings, close collaboration, and the input of many subject matter experts in order to meet the rolling weekly publication deadline and provide relevant, timely information to AZCOVIDTXT subscribers. Thus, developing health messaging material continuously for a weekly publication deadline was a challenge for the team. This task was made even more difficult by the rapidly evolving COVID-19 science as well as national and Arizona-specific guidance.

Another challenge encountered was developing effective health promotion messages with a 160-character limit for both English and Spanish SMS. The general idea for the message would be refined down to a single idea or action, then reduced to as few words as possible while remaining grammatically correct and understandable.

There are limitations to the messaging component of this project. The structure of the system only allowed for SMS to be sent to the phone number of the single participating household member. However, the social media and website posts could be accessed regularly by any Arizonan, regardless of whether they had subscribed to AZCOVIDTXT. In addition, as of July 2021, the subscriber base was largely white and non-Hispanic and thus was not representative of the Arizona population. Potential ideas for promoting the service to a more diverse audience include adding communication modalities (e.g., utilizing radio or printed materials), targeting messaging more granularly to vulnerable populations, and utilizing translational services to offer health information in additional languages (13). We are engaging in efforts to expand this demographic reach in order to better serve Arizona communities. Finally, this project was rapidly developed in real time at the outset of an unprecedented global health threat. As a result, it was not driven by the goal of theory testing or with evaluation in mind as might be the case in formal health interventions. As researchers work to prepare for the next pandemic, however, it would be worthwhile to start from one or more established theoretical frameworks such as those involving technology adoption or design science.

### Practice implications

The project's successes and challenges helped to identify several of AZCOVIDTXT's programmatic elements that were essential to the system's effectiveness and efficiency. These implications were informed through participant feedback and AZCOVIDTXT staff input.

#### Addressing community-specific needs

Tailoring content to identified needs of the community will likely improve user retention and increase engagement. This is consistent with previous research on health messaging and aligns with feedback provided by our subscribers (14).

#### Timeliness of content

Due to the nature of continually evolving COVID-19 research, it is essential that content be developed and released in a timely manner to ensure its' relevance to readers. Users noted being more actively engaged with messaging when content was “relevant and actionable.”

#### Program adaptability

Similarly, it is important that the program operations and logistics (e.g., website and survey structure) remain flexible to accommodate changes as updates are made to COVID-19 guidance or as the community needs evolve. This was informed through team experiences and multiple survey iterations to account for the intake of continually changing community challenges.

#### Automation of processes

Automating project aspects such as SMS dissemination and tailoring, or the release of regular community feedback opportunities will reduce the burden on project staff.

#### Bidirectional information exchange

Engaging the audience in directing the content of the public health messaging successfully identified theme areas. Adding contextualization for messaging that is relevant to users is a proposed strategy for disseminating tailored content (14).

#### Multiple modalities to reach a wider audience

Having redundancies of different lengths (SMS vs. article), graphical vs. word (infographic vs. article) allowed messaging to reach multiple preferences and audience members.

This project built the foundation for continued work for Arizona health messaging, such as expanding to information about other emerging health issues (e.g., West Nile virus, extreme heat, air quality, fires and extreme weather events). Future directions of AZCOVIDTXT include better outreach and engagement aimed at vulnerable populations (e.g., racial and ethnic minority groups, rural communities, etc.), automating content identification through machine learning, and providing more regular opportunities for user feedback.

## Data availability statement

The original contributions presented in the study are included in the article/Supplementary material, further inquiries can be directed to the corresponding author.

## Author contributions

SR, KE, SI, TC, MO, NM, and MS served as project leadership. PC, SF, JB, and MY developed and disseminated health messaging content. SR and KE reviewed all health messaging content. All parties contributed to protocol development. PC, SF, and SR wrote the manuscript. All authors contributed edits to the final manuscript.

## Conflict of interest

The authors declare that the research was conducted in the absence of any commercial or financial relationships that could be construed as a potential conflict of interest.

## Publisher's note

All claims expressed in this article are solely those of the authors and do not necessarily represent those of their affiliated organizations, or those of the publisher, the editors and the reviewers. Any product that may be evaluated in this article, or claim that may be made by its manufacturer, is not guaranteed or endorsed by the publisher.

## Supplementary material

Supplemental Figure 1 AZCOVIDTXT Website.

Supplemental Figure 2 Interactive map used to tailor messaging.

Supplemental Figure 3 Social Media Infographic Examples (in English and Spanish).

## Abbreviations

AZCOVIDTXT: Arizona COVID Text
SMS: Short message service.
