# Supplementary figures and images for: Design and implementation of a health messaging protocol employed for use within a COVID-19 health dissemination platform
Source: Front Public Health. 2022 Nov 24;10:942795. doi: 10.3389/fpubh.2022.942795 (PMC9731134; doi:10.3389/fpubh.2022.942795)

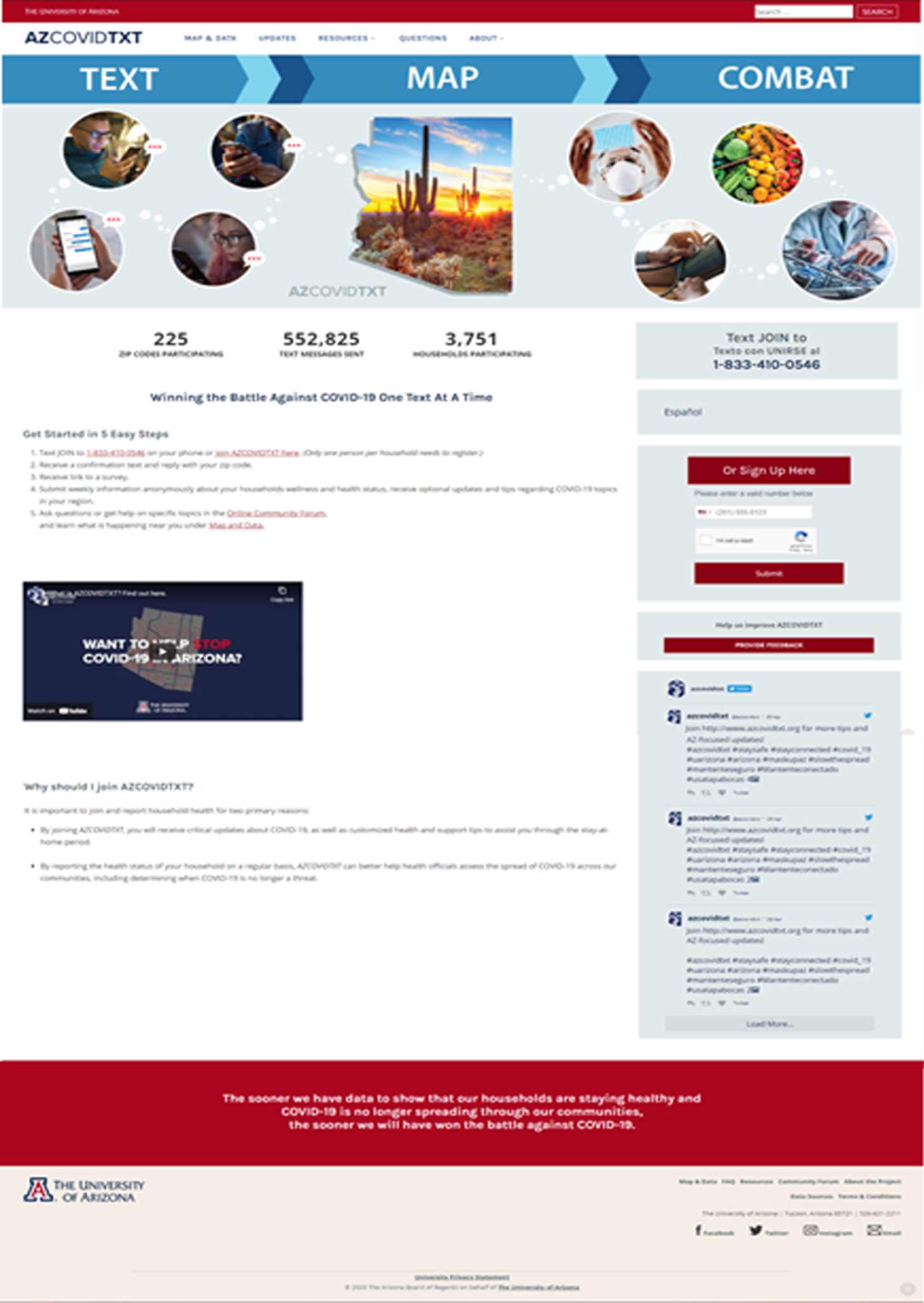

Supplement: Supplemental Figure 1 — AZCOVIDTXT Website. [file Image_1.TIFF]

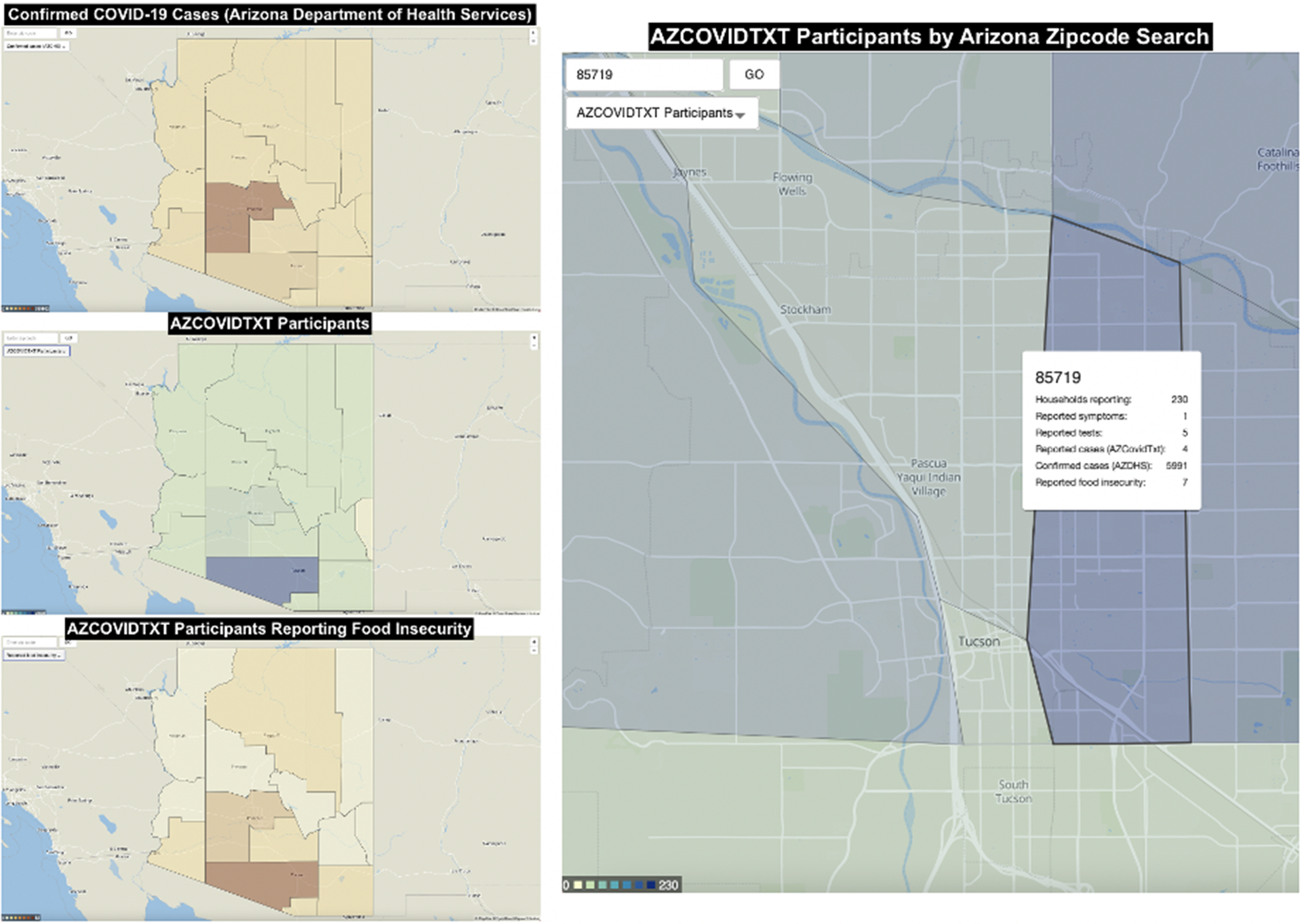

Supplement: Supplemental Figure 2 — Interactive map used to tailor messaging. [file Image_2.TIFF]

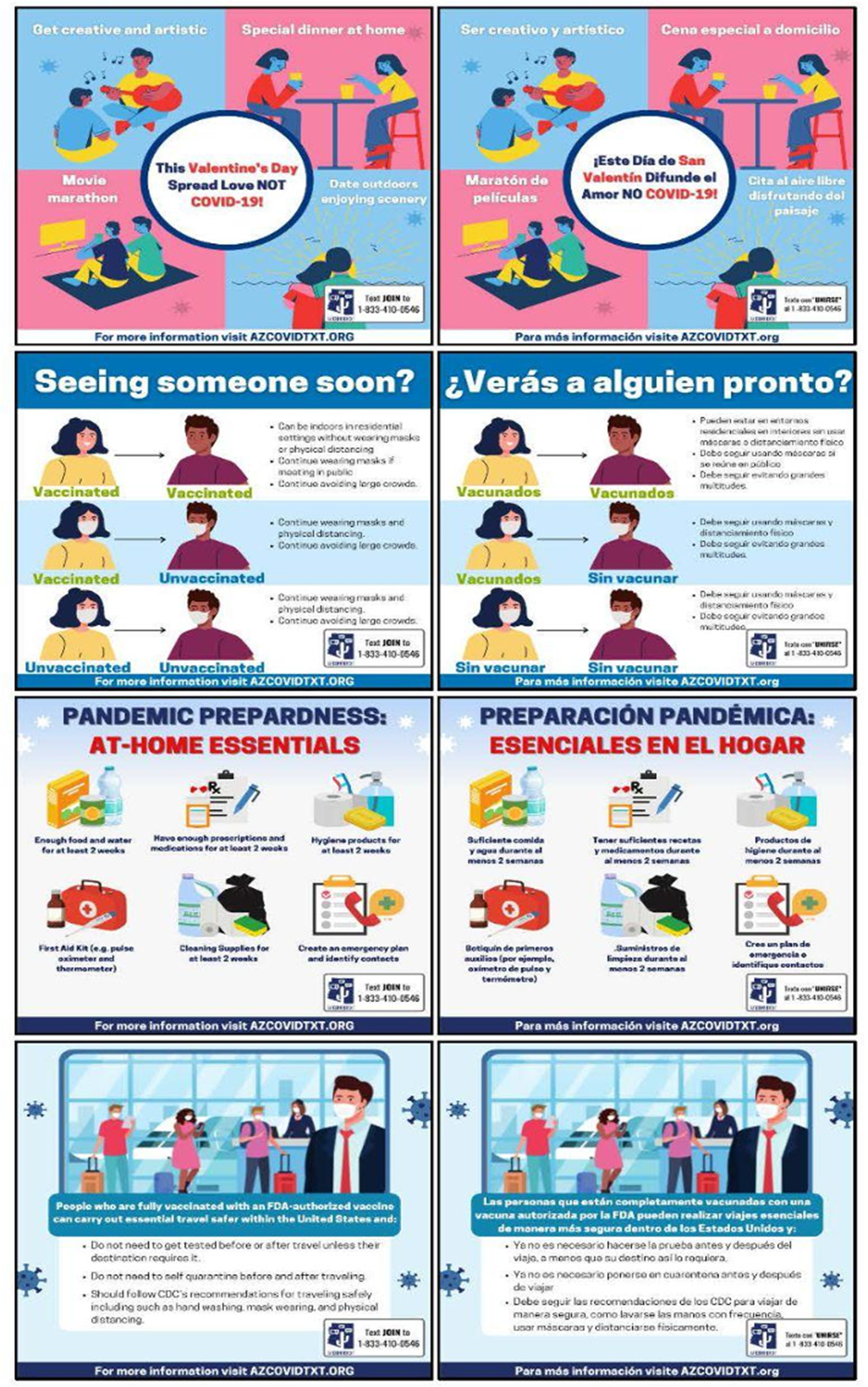

Supplement: Supplemental Figure 3 — Social Media Infographic Examples (in English and Spanish). [file Image_3.TIFF]
